# A completeness indicator of gestational and congenital syphilis information in Brazil

**DOI:** 10.11606/s1518-8787.2023057004789

**Published:** 2023-07-18

**Authors:** Guilherme Lopes de Oliveira, Andrêa JF Ferreira, José Guilherme Santana, Raquel Martins Lana, Andrey Moreira Cardoso, Carlos Teles, Rosemeire L. Fiaccone, Rosana Aquino, Maria Auxiliadora Santos Soares, Enny S. Paixao, Idália Oliveira Santos, Leonardo Salvi, Maurício L. Barreto, Maria Yury Ichihara

**Affiliations:** I Fundação Oswaldo Cruz Centro de Integração de Dados e Conhecimentos para Saúde Salvador BA Brasil Fundação Oswaldo Cruz. Centro de Integração de Dados e Conhecimentos para Saúde, Salvador, BA, Brasil; II Centro Federal de Educação Tecnológica de Minas Gerais Departamento de Computação Belo Horizonte MG Brasil Centro Federal de Educação Tecnológica de Minas Gerais. Departamento de Computação. Belo Horizonte, MG, Brasil; III Drexel University Dornsife School of Public Health The Ubuntu Center on Racism, Global Movements & Population Health Equity Philadelphia USA Drexel University. Dornsife School of Public Health. The Ubuntu Center on Racism, Global Movements & Population Health Equity. Philadelphia, USA; IV Centro Nacional de Supercomputación Barcelona España Centro Nacional de Supercomputación. Barcelona, España; V Fundação Oswaldo Cruz Escola Nacional de Saúde Pública Sérgio Arouca Department of Endemic Diseases Samuel Pessoa Rio de Janeiro RJ Brasil Fundação Oswaldo Cruz. Escola Nacional de Saúde Pública Sérgio Arouca, Department of Endemic Diseases Samuel Pessoa. Rio de Janeiro, RJ, Brasil; VI Universidade Estadual de Feira de Santana Departamento de Ciências Exatas Feira de Santana BA Brasil Universidade Estadual de Feira de Santana, Departamento de Ciências Exatas. Feira de Santana, BA, Brasil; VII Universidade Federal da Bahia Instituto de Matemática e Estatística Salvador BA Brasil Universidade Federal da Bahia. Instituto de Matemática e Estatística, Salvador, BA, Brasil; VIII Universidade Federal da Bahia Instituto de Saúde Coletiva Salvador BA Brasil Universidade Federal da Bahia. Instituto de Saúde Coletiva. Salvador, BA, Brasil.; IX London School of Hygiene and Tropical Medicine London United Kingdom London School of Hygiene and Tropical Medicine. London, United Kingdom

**Keywords:** Health Information Systems, Syphilis, Congenital, Data Accuracy

## Abstract

**OBJECTIVE:**

To evaluate the quality of information on gestational syphilis (GS) and congenital syphilis (CS) on the *Sistema de Informação de Agravos de Notificação* (SINAN-Syphilis Brazil – Notifiable Diseases Information System) by compiling and validating completeness indicators between 2007 and 2018.

**METHODS:**

Overall, care, and socioeconomic completeness scores were compiled based on selected variables, by using *ad hoc* weights assigned by experts. The completeness scores were analysed, considering the region and area of residence, the pregnant woman’s race/colour, and the year of case notification. Pearson’s correlation coefficients were used to validate the scores obtained by the weighted average method, compared with the values obtained by principal component analysis (PCA).

**RESULTS:**

Most selected variables presented a good or excellent degree of completeness for GS and CS, except for clinical classification, pregnant woman’s level of education, partner’s treatment, and child’s race/colour, which were classified as poor or very poor. The overall (89.93% versus 89.69%) and socioeconomic (88.71% versus 88.24%) completeness scores for GS and CS, respectively, were classified as regular, whereas the care score (GS-90.88%, and CS-90.72%) was good, despite improvements over time. Differences in the overall, care and socioeconomic completeness scores according to region, area of residence, and ethnic-racial groups were reported for syphilis notifications. The completeness scores estimated by the weighted average method and PCA showed a strong linear correlation (> 0.90).

**CONCLUSION:**

The completeness of GS and CS notifications has been improving in recent years, highlighting the variables that form the care score, compared with the socioeconomic scores, despite differences between regions, area of residence, and ethnic-racial groups. The weighted average was a viable methodological alternative easily operationalised to estimate data completeness scores, allowing routine monitoring of the completeness of gestational and congenital syphilis records.

## INTRODUCTION

Problems with the quality of data registered on Brazilian Health Information Systems (HIS) remain, despite the undeniable progress made in recent years^1^. Different parameters have been used to evaluate the quality of health information, including completeness^2,3^, defined as the degree of variable completion on HIS, a thermometer of the quality of information generated by these systems^3^.

The incompleteness of information from HIS impairs the accuracy of records, affecting the reliability of the information, the adequate knowledge of the health-disease process, and the epidemiological patterns of events^4,5^. It reduces the capacity to plan appropriate actions to prevent and control disease and disorders within the scope of epidemiological surveillance, integrated into those of care^6^. Therefore, HIS completeness is essential to promote and protect the population’s health and to appropriately plan and manage resources, including investments for the continuous improvement of these systems by the Ministry of Health^2,7^.

Syphilis is among the diseases of great relevance in public health, and, therefore, we need qualified information for its monitoring. As a secular and historically neglected disease, syphilis is caused by the bacterium *Treponema pallidum* and transmitted mainly sexually and vertically^8,9^. The World Health Organization (WHO) estimates that approximately 12 million new syphilis cases occur annually, mainly in low-and-middle-income countries^9^. During the pregnancy, labour, and postpartum phases, the mother-baby binomial has been the focus to track maternal syphilis in prenatal care, preventing the vertical transmission of congenital syphilis (CS).

In Brazil, approximately 61,441 cases of gestational syphilis (GS) and 22,065 cases of CS were reported in 2021, with a GS detection rate of 20.8 and CS incidence rate of 8.9 per 1,000 live births^10^. The CS was only included on the national list of diseases requiring mandatory notification in 1986, GS and acquired syphilis were added in 2005 and 2010, respectively^6,11^, and are reported with individual notification records (INR) on the *Sistema de Informação de Agravos de Notificação* (SINAN – Notifiable Diseases Information System). Filling INR fields, including those not mandatory, is recommended following confirmation of a syphilis case^6^. Analysing the information generated by GS and CS notification records is fundamental to monitoring diseases indicators and ensuring the improvement of prevention, control, and care, besides indicating strategies to enhance its completion quality.

Few national studies have evaluated the completeness of SINAN-Syphilis information^7,12,13^ and the factors that influence its variation, such as region, year of notification, area of residence, and race/colour of the individual, as has been evidenced in other studies with HIS in Brazil^2,7,14^. Valuable indicators that are easily operationalised, which enable health managers and professionals to monitor the completeness of the information registered on HIS are also lacking. This study aims to evaluate the quality of the information by compiling and validating SINAN-Syphilis completeness indicators in Brazil between 2007 and 2018.

## METHODS

This descriptive and analytical study evaluated the degree of completeness of the variables in the GS and CS records available on the Ministry of Health SINAN-Syphilis between 2007 and 2018^6^. We build binary indicators and completeness scores for the overall variables selected and for those classified as socioeconomic or related to care. Completeness degree was classified according to Romero and Cunha^15^ as follows: excellent (> 95%), good (90%–95%), regular (80%–90%), poor (50%–80%), and very poor (≤ 50%).

The following variables were selected after a literature review of social determinants and factors related to the prevalence and incidence of gestational and congenital syphilis cases^16^ and their importance for syphilis surveillance^7,9,10^, monitoring^4,12^, and the knowledge of its epidemiological profile in the Brazilian territory^7,20^: **GS** – pregnant woman’s age range, area of residence, level of education, race/colour, gestational age, qualitative results of treponemal and non-treponemal tests (VDRL), treatment regime, clinical classification, and partner’s treatment; and **CS** – age range, child’s sex and race/colour, area of residence, mother’s level of education and race/colour, qualitative VDRL results for the child, case evolution, attendance to prenatal care, treatment regime, time of diagnosis, and partner’s treatment.

### Building the Completeness Indicators and Scores

**(a) Binary completeness indicator:** to evaluate the individual completion of each variable, the proportion of individual notification records with complete information was obtained, considering the indicator of the presence of information (1) or of it being absent, ignored, or inconsistent (0) in the selected variables.

**(b) Completeness scores:** to evaluate the completion of a set of variables, the weighted average method was used, based on the values of the binary indicators defined in (a) and weights defined on a scale from 1 to 4, varying from the lowest to the highest importance, related to syphilis surveillance, epidemiology, and disease control. Public health specialists and health professionals working in the Brazilian Unified Health System (SUS, in Portuguese Sistema Único de Saúde) assigned *ad hoc* weights for each variable. Subsequently, these subjective weights were evaluated and agreed upon by the study researchers, considering the value endorsed by most of the specialists (Table 1). Note that this approach is commonly used to propose indexes and tends to present similar results when compared with other weighting methods^21^. Based on the final weights, the overall score considers all variables selected, whereas the socioeconomic and care scores consider the variables classified as socioeconomic and care, respectively. The three scores vary between 0 and 1, and the higher the score value, the better the completeness of the set of variables considered. The completeness scores were analysed according to the year of case notification, region, area of residence, and pregnant woman’s race/colour.

**Table 1:** Analysis of the overall, socioeconomic, and care variables and scores completeness. SINAN-Syphilis, 2007–2018.

Variables	Congenital syphilis		Gestational Syphilis	
	
	Completeness	Weights	Completeness	Weights
	
	n (%)		n (%)	
Socioeconomic				
Pregnant woman’s level of education	141,563 (71.51)	4	218,457 (71.44)	4
Pregnant woman’s race/colour	177,000 (89.41)	4	279,863 (91.51)	4
Pregnant woman’s age range	191,947 (96.96)	3	305,794 (99.99)	3
Area of residence	190,281 (96.11)	3	295,881 (96.75)	3
Child’s race/colour	158,055 (79.84)	2	-	-
Child’s age range	197,922 (99.97)	2	-	-
Sex	186,368 (94.14)	1	-	-
Socioeconomic score	88.24	-	88.71	-
Care				
Partner’s treatment	156,672 (79.14)	4	89,004 (29.12)	-
Treponemal test	172,673 (87.22)	4	276,013 (90.25)	4
Treatment regime	176,192 (89.00)	4	290,434 (94.97)	4
Attended prenatal care	185,884 (93.89)	4		
Non-treponemal test (VDRL)			290,009 (94.83)	4
Time of diagnosis	187,688 (94.80)	4		
Clinical classification			276,013 (72.10)	3
Gestational age			305,213 (99.80)	3
Non-treponemal test (VDRL)	191,435 (96.70)	4		
Evolution	188,956 (95.44)	3	-	-
Care score	90.72	-	90.88	-
Overall Score	89.69	-	89.93	-

* Partner’s treatment was not used to calculate the GS score

### Validating the Completeness Scores

The validation was based on comparing the scores obtained by the weighted average method with those obtained with principal component analysis (PCA), a multivariate statistical technique used to reduce dimensionality, generate indexes (scores), and cluster individuals or variables. The tetrachoric correlation matrix of the binary completeness indicators was used to extract the principal components^24^. The binary indicators of the variables with excellent completeness (> 95%) were not considered in the analysis due to the low variability between the categories, and the “partner’s treatment” variable was disregarded in the CS analysis due to the high percentage of data either missing or ignored. We calculated the PCA scores for each record using the regression method^25^. The mean of scores associated with the first two principal components extracted in the analysis was considered for the overall score. For socioeconomic and care scores, only the first principal component was considered. Lastly, we analyse the relationship between scores calculated by the weighted average and PCA methods via scatter plots and estimate Pearson’s linear correlation coefficient. The R software, version 3.6.0, and the psych package were used in the PCA^26^.

### Ethical Aspects

This study used anonymised administrative health data provided by the Ministry of Health, exempting submission to the Ethics and Research Committee.

## RESULTS

A total of 305,891 GS and 197,974 CS cases were notified between 2007 and 2018, registering an increase of 9.1 and 5.3 times, respectively, in notification cases in 2018. Most GS notifications were from the Southeast (44.8%) and Northeast (21.3%) regions, followed by the South (14.4%), North (10.8%), and Midwest (8.6%). The CS showed a similar pattern. Considering the area of residence of GS notifications, 88.3% occur in the urban area, 7.7% in rural, 0.7% from the peri-urban area, and 3.2% of the notifications did not fill out this information. Most CS notifications (89.3%) were from urban areas, 6% from rural, and 0.8% from peri-urban areas, and 3.9% of the cases did not include this information. The prevalence of GS cases was concentrated among women who self-declared as mixed-race (48.3%), white (29.3%), black (12.3%), Asian descendant/yellow (0.9%), and indigenous (0.8%), with race/colour information being absent in 8.5% of the notifications. For CS, 48.9% of case notifications was mixed-race, 25.4% white, 5.0% black, 0.4% indigenous, 0.24% yellow, and 20.1% of notifications lacked this information.

Analysis of the completeness of GS records, measured by the binary indicator (Table 1), was classified as excellent (> 95%) for the pregnant woman’s age range, area of residence, and gestational age; good (90%–95%) for the pregnant woman’s race/colour, qualitative results of treponemal test, VDRL test, and treatment regime; poor (50%–80%) for the pregnant woman’s level of education and clinical classification; and very poor (< 50%) for the partner’s treatment information. The CS notification records presented excellent completeness (> 95%) for the mother and child’s age range variable, area of residence, qualitative results of VDRL test, and clinical evolution; good (90%–95%) for child’s sex, prenatal care, and the time of diagnosis; regular (80%-90%) for the mother’s race/colour, treponemal test and treatment regime; and poor (50%–80%) for the mother’s level of education, child’s race/colour, and partner’s treatment.

Moreover, overall, and socioeconomic completeness scores for GS (89.93% and 88.7%) and CS (89.69% and 88.71%) were classified as regular (Table 1), whereas the care score was good (GS – 90.88% and CS – 90.72%) over the years.

**Figure 1:**
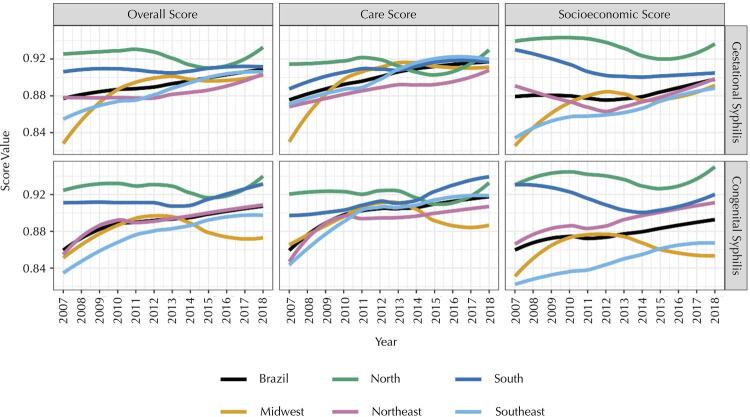
Analysis of completeness scores according to the year of the case notifications and the Brazilian regions, SINAN-Syphilis, 2007-2018.

Between 2007 and 2015 in Brazil (Figure 1), the overall completeness score was evaluated as regular for GS (87.7% in 2007 to 89.9% in 2009) and CS (85.5% in 2007 to 89.9% in 2014) and good from 2016 onwards. For both diseases, the socioeconomic score was also classified as regular over the years, with a growing tendency to improve completeness between 2011 and 2017 for GS (87.5% and 89.8%), and between 2007 and 2017 for CS (85.5% and 89.4%). The care score was classified as regular for GS between 2007 and 2012 and good in the following years, whereas for CS, it remained regular until 2015, with a gradual increase in the completeness scores in the subsequent years. We highlighted that, from 2008, the care score was higher than the socioeconomic score for both diseases. The North and South Brazilian regions presented, over time, improvement in completeness scores for GS and CS, especially for socioeconomic scores (Figure 1). This improvement occurred in the Midwest and Southeast regions for GS, whereas for CS, it was more evident in the Southeast and Northeast regions.

**Figure 2:**
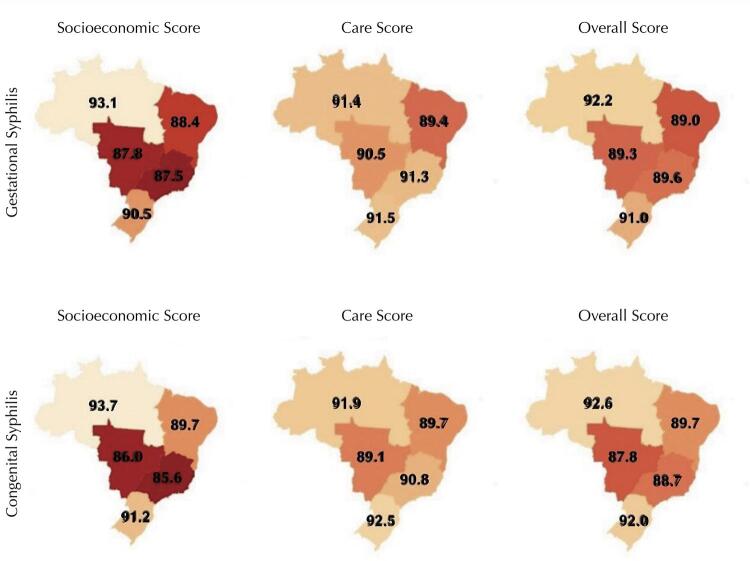
Analysis of completeness scores according to the region of residence for gestational and congenital syphilis, SINAN-Syphilis, 2007-2018.

Note the regional differences of the gestational and congenital completeness scores (Figure 2): the overall and socioeconomic scores were classified as good in the North (GS – 92.2% and CS – 92.6% and GS – 93.1% and CS – 93.7%, respectively) and South regions (GS – 91% and GS – 90.5% and CS – 92% and CS – 91.2%, respectively), and regular in the other regions; the care score for GS was classified as good in all regions (North – 91.4%; Midwest – 90.5%; Southeast – 91.3%; and South – 91.5%), except for the Northeast region (89.4%), where it was regular; for CS, the care score was classified as good for the North (91.9%), Southeast (90.8%), and South regions (92.5%) and regular for the Northeast (89.7%) and Midwest (89.1%) regions.

The numerical values correspond to the score value observed in each region. The darker scores correspond to the lowest completeness scores and the lightest scores to the highest ones.

**Figure 3:**
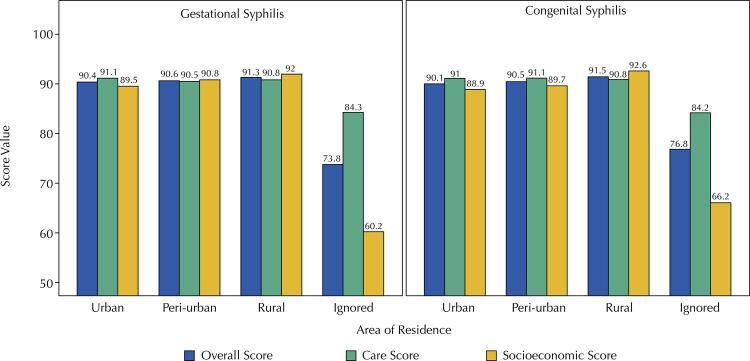
Analysis of completeness scores according to the area of residence of pregnant women for gestational and congenital syphilis, SINAN-Syphilis, 2007-2018.

Gestational and congenital syphilis cases presented information for the area of residence in 96.8% and 96.1% of notification records, respectively (Figure 3). The overall and care scores classifications were good in the urban (GS – 90.4% and 91.1%; CS – 90.1% and 91%), rural (GS – 91.3% and 90.8%; CS – 91.5% and 90.8%) and peri-urban area of residence (GS – 90.6% and 90.5%; CS – 90.5% and 91.1%). However, the socioeconomic score presented a good quality for GS in rural (92.0%) and peri-urban areas of residence (90.8%) but regular in the urban area (89.5%). For CS, the socioeconomic score was good for the rural area (99.2%) and regular for the other areas of residence (urban – 88.9% and peri-urban – 89.7%). However, when information about the area of residence was ignored (GS – 3.2% and CS – 3.9%), the classification of completeness scores worsened (overall and care scores were poor, whereas the socioeconomic score was regular).

**Figure 4:**
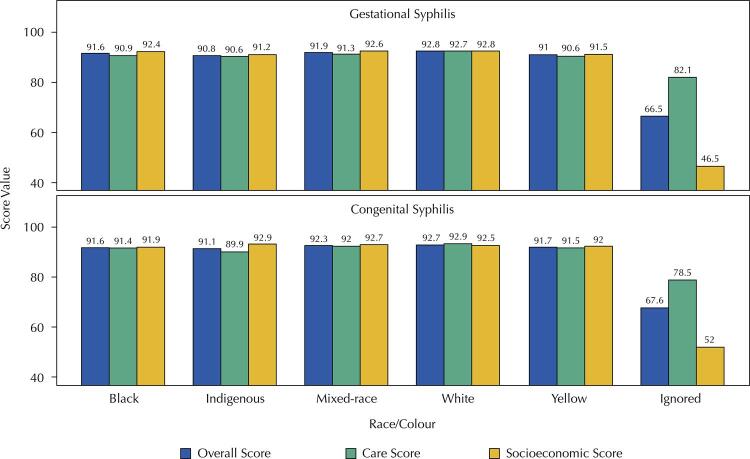
Analysis of completeness scores according to the pregnant woman’s race/colour for gestational and congenital syphilis, SINAN-Syphilis, 2007-2018.

Considering 91.5% and 79.85% of the GS and CS cases (Figure 4), respectively, had race/colour information, we observed that the overall, socioeconomic, and care completeness scores for GS were classified as good for all the racial categories (black, mixed-race, indigenous, yellow, and white). For CS, the overall and socioeconomic scores presented good completeness for all the racial categories. In cases where race/colour information was ignored (GS – 8.5% and CS – 20.15%), all the completeness scores were classified as poor, except the socioeconomic score of GS records, which was regular.

**Figure 5:**
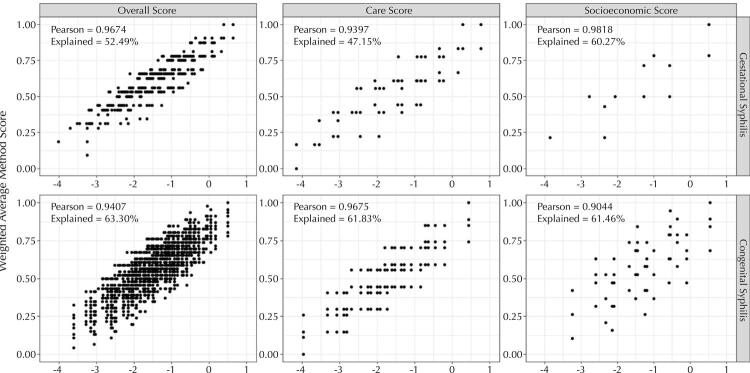
Comparison between the completeness scores obtained by the weighted average method and those obtained by principal component analysis, SINAN-Syphilis, 2007-2018. We have the GS comparisons on the upper line and the CS results on the lower line. The comparative graphs for the socioeconomic scores are in the left-hand column; those of the care scores in the central column; and those of the overall scores in the right-hand column. The graphs present Pearson’s linear correlation coefficient (Pearson’s R). The explanation percentage obtained in the PCA is shown on the horizontal axis of the graphs.

Validation of the overall, socioeconomic and care scores were achieved by comparing them using the weighted average and PCA methods, and the result presented a strong linear correlation (> 0.90) in the scatter plots (Figure 5). In the case of PCA, the explanation percentage for the principal components retained in the analysis varied between 47.15% and 63.30% for the six different scores calculated. The result obtained by both methods was similar.

## DISCUSSION

The results indicate a good or excellent degree of completeness for most of the SINAN-Syphilis variables included in gestational and congenital syphilis notification records, except for clinical classification, pregnant mother’s level of education, partner’s treatment, and child’s race/colour, which were classified as poor or very poor. The overall and socioeconomic completeness scores were similar for GS and CS records, both classified as regular, whereas the care score was classified as good and improved during the period studied. However, differences in scores were verified according to region, area of residence, and ethnic-racial groups. These differences were also observed in studies that evaluated the completeness of information on GS and CS notification cases in Latin American and Caribbean countries^27^. This situation emphasised the need to improve the capacity of countries to collect high-quality data for diseases of public health interest^7,27^.

The GS and CS notifications have increased more than 9 and 5 times, respectively, in recent years, possibly due to the expansion of primary health care coverage, in particular the Family Health Strategy, and the Bolsa Familia Program, which conditions receiving the benefit to the attendance of pregnant women to prenatal care and children to medical appointments to monitor growth and development^14,28^. Improving case detection has not been followed by increasing the completeness of information, with significant regional differences across Brazilian regions, which corroborate previous results described in the literature for other infectious diseases^12,14,18^. Problems with information completeness of GS and CS records may be related to the differences in the commitment level of health professionals regarding the adequate registration of case notification records, the reduced number of health professionals in health services to complete a significant number of records, and the non-prioritisation of syphilis by health managers^7,12^. Considering that, often, notification is seen as a bureaucratic activity of secondary importance, training health professionals on the need to generate quality information for epidemiological surveillance is essential to ensure the integrated flow of information between the responsible sectors, which can reduce differences at the area of residence and regional level in the quality of the information available in syphilis notification records^5,7,29^.

The care score completeness for CS was classified as regular for the indigenous group and good for the other racial categories, indicating possible difficulties in the monitoring of indigenous children and, consequently, in the adequate case notification, in addition to the absence or insufficiency in the training of indigenous health teams^30^. Many factors may contribute to information incompleteness, such as the availability of time to complete the INR fields, considering the demands of the service, the existence of linguistic and cultural barriers, and difficulty in entering data onto SINAN by the base-centres and/or Special Indigenous Sanitary Districts, since, besides not being notifying units, they do not interact with those in the municipal health system^13,30^.

Note that the “partner’s treatment” variable had a poor and very poor degree of completeness for GS and CS, respectively. We supposed that this occurs due to the Brazilian Ministry of Health modifying the definition of CS cases by including the criterion “adequate treatment of the pregnant woman” and disregarding the information on the simultaneous “partner’s treatment” since September of 2017. Therefore, this variable has become a complementary and non-mandatory filling field in INR^31^. Insufficient completeness of the field “concomitant treatment of the partner,” influenced by the non-mandatory registration of the information, harms the monitoring of syphilis cases, mainly CS, which reduces the possibility to assess the real transmission scenario of syphilis and the quality of prenatal care^12,32,33^. Studies indicate that the incompleteness of SINAN information, particularly for CS, and the high underreporting of cases limit the use of this system in studies of incidence and factors related to the growth of syphilis in Brazil^7,19,34^.

Moreover, many variables present in INR are considered important markers for the monitoring and epidemiological evolution of GS and CS in the country (i.e., partner’s treatment for CS) and support the adoption of therapeutic conduct of the disease (i.e., clinical classification and time of diagnosis of the disease). Other fields, such as the mother’s level of education and race/colour of the mother or child, are used as markers of social and racial inequalities related to infection and/or associated negative outcomes^4,7,19^, which highlighted the importance to improve quality data in SINAN-Syphilis. Also, we should highlight that the incompleteness of information related to education level had been observed in several HIS throughout the national territory, limiting its use in research^35^.

Data incompleteness directly reflects on the quality of information by not demonstrating the reality of the epidemiological profile of syphilis cases notified in the country^36^. This scenario complicates the planning of strategies and actions to prevent and control syphilis, contributes to the inefficiency of public policies to tackle the disease, and interferes in achieving the goals of eliminating and eradicating syphilis cases proposed by the WHO^9,14,36^. This situation becomes more complex when we consider regional, residential, and ethnic-racial variations in the information completeness scores in a country of continental dimensions, such as Brazil. Therefore, we suggest that future studies analyse the factors associated with the incompleteness of data and its variations among different groups and regions, which will allow the adoption of strategies to reduce differentials in the quality of SINAN-Syphilis information.

Our results indicate an improvement in the degree of completeness of SINAN-Syphilis information during the study period, despite the observed variations. We present a proposal to evaluate the quality of SINAN-Syphilis information, which is still little explored in the national scenario, building scores based on the weighted average of completeness indicators that are easily operationalised. There are some limitations related to defining weights for the weighted average method, based on the experience of specialists and not by the correlation structure from the data obtained by the PCA. However, weighting is commonly used to compile health indicators and, in our case, produced results consistent with those obtained via PCA. This study only accessed the completeness of information, one of the parameters used to evaluate the quality of the information, not considering cases of underreporting or agreement of information, which are also important parameters to be considered when we evaluate the quality of HIS^3^.

## CONCLUSION

The completeness of GS and CS notifications has improved in recent years, mainly with the care score variables, compared with the socioeconomic variables, despite differences between Brazilian regions, ethnic-racial groups, and area of residence for both diseases. However, essential information to describe the epidemiological profile and monitor GS and CS still have poor completeness, compromising the planning of epidemiological surveillance and health care actions. We recommended promoting health teams’ training and health managers adopting strategies aiming at improving the complete filling of INR, given the prioritisation of other demands in health services. Lastly, health professionals and managers can incorporate indicators of easy compilation and interpretability for monitoring the completeness of SINAN-syphilis data, as proposed in this study, into the routine of evaluating the quality of information aiming at its improvement.

## References

[1] Laguardia J, Domingues CM, Carvalho C, Lauerman CR, Macário E, Glatt R (2004). Sistema de informação de agravos de notificação em saúde (Sinan): desafios no desenvolvimento de um sistema de informação em saúde. Epidemiol Serv Saude.

[2] Marques CA, Siqueira MM, Portugal FB (2020). Assessment of the lack of completeness of compulsory dengue fever notifications registered by a small municipality in Brazil. Cien Saude Colet.

[3] Lima CR, Schramm JM, Coeli CM, Silva ME (2009). Review of data quality dimensions and applied methods in the evaluation of health information systems. Cad Saude Publica.

[4] Saraceni V, Domingues RM, Vellozo V, Lauria LM, Dias MAB, Ratto KMN (2007). Surveillance of syphilis in pregnancy. Epidemiol Serv Saude.

[5] Correia LO, Padilha BM, Vasconcelos SM (2014). Methods for assessing the completeness of data in health information systems in Brazil: a systematic review. Cien Saude Colet.

[6] Ministério da Saúde (BR) (2006). Sistema de Informação de Agravos de Notificação-SINAN: normas e rotinas.

[7] Saraceni V, Pereira GF, Silveira MF, Araujo MA, Miranda AE (2017). Epidemiological surveillance of vertical transmission of syphilis: data from six federal units in Brazil. Rev Panam Salud Publica.

[8] Hook EW (2017). Syphilis. Lancet.

[9] World Health Organization (2018). Report on global sexually transmitted infection surveillance.

[10] Ministério da Saúde (BR), Departamento de Doenças de Condições Crônicas e Infecções Sexualmente Transmissíveis (2022). Boletim Epidemiológico de Sífilis 2021.

[11] Dantas LA (2017). [Epidemiologic profile of acquired syphilis diagnosed and notified at a maternal-child university hospital]. Enferm Glob.

[12] Soares MA, Aquino R (2021). Completeness and characterization of gestational syphilis and congenital syphilis records in Bahia, Brazil, 2007-2017. Epidemiol Serviços Saúde.

[13] Braz RM, Oliveira PT, Reis AT, Machado NM (2013). [Evaluation of the race/color variable completeness in the national health information systems for the measuring of ethnic-racial inequality in indicators used by the Performance Index of the Brazilian Unified Health System]. Saúde Debate.

[14] Saraceni V, Miranda AE (2012). Coverage by the Family Health Strategy and diagnosis of syphilis in pregnancy and congenital syphilis. Cad Saude Publica.

[15] Romero DE, Cunha CB (2006). Quality of socioeconomic and demographic data in relation to infant mortality in the Brazilian Mortality Information System (1996/2001). Cad Saude Publica.

[16] Domingues RM, Leal MC (2016). [Incidence of congenital syphilis and factors associated with vertical transmission: data from the Birth in Brazil study]. Cad Saude Publica.

[17] França IS, Batista JD, Coura AS, Oliveira CF, Araújo AK, Sousa FS (2015). Factors associated to the notification of congenital syphilis: an indicator of quality of prenatal care]. Rev Rene.

[18] Conceição HN, Câmara JT, Pereira BM (2020). [Epidemiological and spatial analysis of cases of gestational and congenital syphilis]. Saúde Debate.

[19] Benzaken AS, Pereira GF, Cunha AR, Souza FM, Saraceni V (2019). Adequacy of prenatal care, diagnosis and treatment of syphilis in pregnancy: a study with open data from Brazilian state capitals. Cad Saude Publica.

[20] Cardoso AR, Araújo MA, Cavalcante MD, Frota MA, Melo SP (2018). Analysis of cases of gestational and congenital syphilis between 2008 and 2010 in Fortaleza, State of Ceará, Brazil. Cien Saude Colet.

[21] Nguefack‐Tsague G, Klasen S, Zucchini W (2011). On weighting the components of the human development index: a statistical justification. J Human Dev Capabil.

[22] Hagerty MR, Land KC (2007). Constructing summary indices of quality of life: a model for the effect of heterogeneous importance weights. Sociol Methods Res.

[23] Chowdhury S, Squire L (2006). Setting weights for aggregate indices: an application to the commitment to development index and human development index. J Dev Stud.

[24] Kirk DB (1973). On the numerical approximation of the bivariate normal (tetrachoric) correlation coefficient. Psychometrika.

[25] MingotI SA (2007). Análise de dados através de métodos de estatística multivariada. uma abordagem aplicada.

[26] Foundation for Statistical Computing (2021). R: The R project for statistical computing.

[27] Serruya SJ, Duran P, Martinez G, Romero M, Caffe S, Alonso M (2015). Maternal and congenital syphilis in selected Latin America and Caribbean countries: a multi-country analysis using data from the Perinatal Information System. Sex Health.

[28] Baroni L, Alves RF, Boccolini CS, Salles R, Gritz R, Paixão B (2021). Database on the coverage of the “Bolsa-Família” conditioning cash-transfer program: Brazil, 2005 to 2021. BMC Res Notes.

[29] Muguande OF, Ferraz ML, França E, Gontijo ED (2011). Evaluation of the quality system of epidemiological surveillance of acute Chagas disease in Minas Gerais, 2005-2008. Epidemiol Serv Saude.

[30] Tiago ZD, Picoli RP, Graeff SV, Cunha RV, Arantes R, Tiago ZS (2017). Underreporting of gestational, congenital and acquired syphilis among indigenous peoples in Mato Grosso do Sul State, Brazil, 2011-2014. Epidemiol Serv Saude.

[31] Ministério da Saúde (BR), Departamento de Doenças de Condições Crônicas e Infecções Sexualmente Transmissíveis (2017). Nota Informativa no 02-SEI/2017 - DCCI/SVS/MS.

[32] World Health Organization (2007). The global elimination of congenital syphilis: rationale and strategy for action.

[33] Ministério da Saúde (BR) (2007). Manual de normas e rotinas do SINAN.

[34] Heringer R (2002). Racial inequalities in Brazil: a synthesis of social indicators and challenges for public policies. Cad Saude Publica.

[35] Lima DJ, Chagas AC, Mendes IC, Oriá MO, Aquino P S, Pinheiro AK (2014). Completeness and consistency of data on notified HIV-positive pregnant women. Rev Enferm UERJ.

[36] Komka MR, Lago EG (2007). Congenital syphylis: notification and reality. Sci Med.

